# Photosynthetic Activities, Phytohormones, and Secondary Metabolites Induction in Plants by Prevailing Compost Residue

**DOI:** 10.3390/metabo14080400

**Published:** 2024-07-24

**Authors:** Lord Abbey, Samuel Kwaku Asiedu, Sparsha Chada, Raphael Ofoe, Peter Ofori Amoako, Stella Owusu-Nketia, Nivethika Ajeethan, Anagha Pradeep Kumar, Efoo Bawa Nutsukpo

**Affiliations:** 1 Department of Plant, Food, and Environmental Sciences, Faculty of Agriculture, Dalhousie University, 50 Pictou Road, Bible Hill, NS B2N 5E3, Canada; sasiedu@dal.ca (S.K.A.); sp950212@dal.ca (S.C.); pt269228@dal.ca (P.O.A.); aj634155@dal.ca (N.A.); anaghapradeepkumar@dal.ca (A.P.K.); bawa.nutsukpo@dal.ca (E.B.N.); 2 Biotechnology Centre, College of Basic and Applied Sciences, University of Ghana, P.O. Box LG 25 Legon, Ghana; sowusu-nketia@ug.edu.gh (S.O.-N.)

**Keywords:** *Solanum lycopersicum*, soil amendment, plant metabolites, plant resilience, plant growth regulators

## Abstract

Compost residue enriches soil health with the potential to enhance plant metabolism and hormonal balance, but has not yet been studied. A study was performed to determine how prevailing compost residue induces tomato (*Solanum lycopersicum* ‘Scotia’) plant morpho-physiology, phytohormones, and secondary metabolites. Plants were grown in soils with a previous history of annual (AN) and biennial (BI) compost amendments. The controls were soil without compost (C) amendment and municipal solid waste compost (MSWC) alone. The MSWC- and AN-plants had similar and significantly (*p* < 0.05) highest growth and photosynthetic activities compared to the BI- or C-plants. Total phenolics and lipid peroxidase activity were significantly (*p* < 0.001) high in BI-plants, while hydrogen peroxide and antioxidant capacity were significantly (*p* < 0.001) high in AN-plants. MSWC-plants recorded the highest cis-abscisic acid, followed by AN-, and then BI- and C-plants. Cis-zeatin, trans-zeatin, and isopentenyladenine ribosides were detected in the MSWC- and AN-plants but not in the BI- or C-plants. Furthermore, gibberellins GA53, GA19, and GA8 were high in the MSWC-plants, but only GA8 was detected in the AN plants and none in the others. Besides, MSWC plants exhibited the highest content of 1-aminocyclopropane-1-carboxylic acid. Conjugated salicylic acid was highest in the BI-plants, while jasmonic acid-isoleucine was highest in MSWC-plants and C plants. In conclusion, prevailing compost chemical residues upregulate plant growth, phytohormones, and metabolic compounds that can potentially increase plant growth and abiotic stress defense. Future work should investigate the flow of these compounds in plants under abiotic stress.

## 1. Introduction

Plants are sessile and are thus, continuously exposed to diverse environmental stress conditions. However, plants possess signaling networks that are capable of immediately detecting external stimuli before activating metabolic responses for adaptation to the new environment [1]. The response to external stimuli involves the regulation of genes, metabolic reprogramming, and the crosstalk of phytohormones. The role of phytohormones in plant growth and development and tolerance to environmental stresses have been widely investigated [2,3]. While auxins, cytokinins, brassinosteroids, gibberellins, and strigolactones have been implicated in major plant growth and development regulations [4], abscisic acid, ethylene, jasmonic acid, and salicylic acid are commonly associated with stress regulation [5,6]. These phytohormones have distinct functions but crosstalk to act synergistically or antagonistically with each other to modulate the biosynthesis of secondary metabolites [7]. Notably, secondary metabolites like polyphenols play a key protective role in the mitigation of photo-oxidative damage in plants by limiting reactive oxygen species produced under abiotic stress conditions [8,9]. However, it is not clear how the biosynthesis and activities of these stress response compounds are influenced by organic soil amendments like compost.

Municipal solid waste compost (MSWC) has become a popular organic soil amendment for improving soil health, crop growth, and productivity due to its rich and stable organic matter content. It is well documented that soil organic matter derived from MSWC plays a crucial role in enhancing soil bio-physicochemical properties [10,11]. Typically, MSWC improves soil structure and enhances soil fertility, soil water retention capacity, soil aeration, and a beneficial soil microbiome that supports plant growth and resilience [10,12,13]. Additionally, Ampese, et al. [14] used a life cycle assessment to demonstrate the environmental benefits of compost, which include reductions in greenhouse gas emissions and carbon footprint. Many studies point to the use of microbiologically rich compost for abiotic stress tolerance in plants [15], but the mechanism of action is not yet understood. Common knowledge is that compost contains humic and non-humic substances that contribute to plant tolerance to abiotic stress. However, the chemical composition of compost can vary depending on the feedstock, process conditions, and stage of maturity. For instance, Klimas, et al. [16] found that the presence or absence of plant hormones such as auxin, gibberellins, and cytokinins in MSWC was dependent on feedstock and conditions during the composting process. Importantly, the impact of compost residue after long-term application on biosynthesis and the abundance of abiotic stress-associated phytohormones and secondary metabolites are unknown.

Generally, the release of nutrients and other compounds from compost occurs at a slow rate, leaving residual nutrients in the soil over time when plant nutrients uptake and nutrient losses are less than the amount added. Previously, our studies revealed enormous differences in soil bio-physicochemical characteristics after five years of MSWC application to soils compared to soils with no added compost [10,17]. Additionally, differences in the frequency of MSWC application differentially altered the metabolic profiles of tested plants [18]. Some specific metabolites, such as amino acids, carnitines, phospholipids, and choline, that were reported are stress-alleviating molecules [18]. Therefore, it was postulated that residual compost may have biostimulatory effects that promote normal cellular balance through homeostasis and alteration of various metabolic pathways to prepare the plants for stressful conditions. As such, it is pertinent to understand the residual compost inducement of phytohormones and secondary metabolites that can be potentially linked to abiotic stress tolerance in plants. The objective of the present study was to determine how the prevailing compost residue will induce tomato (*Solanum lycopersicum* ‘Scotia’) plant morpho-physiology, phytohormones, and secondary metabolites potentially linked to abiotic stress response. The tomato was chosen because it is the most cultivated and consumed vegetable due to its rich and diverse functional bioactive compounds, such as phenolics, flavonoids, polysaccharides, vitamins, and carotenoids, that make it a good model for research [19].

## 2. Materials and Methods

Aspects of the materials and methods section were previously published [10,17,18]. The present study was performed in the Compost and Biostimulant Laboratory in the Department of Plant, Food, and Environmental Sciences, Dalhousie Faculty of Agriculture, Nova Scotia, between August and December 2022.

### 2.1. Field History

The soils used were obtained from 5-year organic field research plots in Aagaard Farms, Brandon, Manitoba, Canada (longitude 99°56′59.99″ W; latitude 49°50′53.99″ N; altitude: 409 m above sea level) between fall 2015 and winter 2020. The soil is of Orthic Black Chernozem solum on moderate to strong calcareous, loamy morainal till of limestone, granitic, and shale origin under the classification of the Newdale series. The soils were treated with ca. 2 t/ha of municipal solid waste compost (MSWC; bulk density was 650 kg/m^3^) obtained from the City of Brandon waste management facility. The MSWC application frequencies were annual (AN) and biennial (BI) applications and a control (C), i.e., no compost application. No synthetic chemical fertilizer or pesticide was used throughout this study. At the end of the 5th year, 20 kg of soil samples were dug randomly with a shovel at a depth of 0–30 cm from five locations per plot and bulked to obtain triplicate sub-samples of 30 kg per treatment. The soils were shipped by next-day courier from the field in Brandon to Dalhousie University in Truro for immediate storage in a walk-in cold room at 4 °C. The residual elemental analyses of the soils after five years are presented in Supplementary Table S1.

### 2.2. Planting and Growth Conditions

The treatments for the pot experiment were the AN-, BI-, and C-soils above with varying MSWC residues, and MSWC alone was used as a treatment instead of soil. Fifteen-cm diameter plastic pots were filled separately with 1 kg of each soil type and placed in plastic saucers. The soils were watered to field capacity and allowed to stand for 24 h before transplanting 5 weeks old tomato ‘Scotia’ seedlings into each pot. The potted-plants were arranged in a completely randomized design with four replications in a Biotronette Mark II Environmental Chamber (Lab-Line Instruments Inc., Melrose Park, IL, USA). The growth chamber was set at 24°/20 °C day/night temperature cycle and 12/12 h day/night light cycle, and plants were watered every 3 days. All the plants were watered to field capacity every other day until the end of the experiment. The potted plants were rearranged weekly to offset unpredictable occurrences due to variations in microclimate.

### 2.3. Data Collection

#### 2.3.1. Plant Growth Parameters and Photosynthetic Activities

The measurement of plant growth and photosynthetic indices (*n* = 9) was performed 6 weeks after transplanting. The plant growth parameters were the number of green leaves, plant height, which was measured from the stem collar to the highest leaf tip with a ruler, and stem diameter, which was measured at 10 cm from the collar with a pair of Vernier callipers (Mastercraft, ON, Canada). Chlorophyll fluorescence indices were determined with an ACM-200+ Anthocyanin meter (Opti-Science Inc., NH, USA). In brief, a clip was fixed to the middle section of a leaf blade and dark-adapted for 25 min before the minimum fluorescence yield (Fo) was measured, followed by the maximum chlorophyll fluorescence (Fm) index when the saturating light pulse was increased from 0 to 3000 μmol m^−2^s^−1^. Variable chlorophyll fluorescence (Fv = Fm − Fo) was recorded. The maximum quantum efficiency of PSII (Fv/Fm) and potential photosynthetic capacity (Fv/Fo) at each saturating pulse were also determined (Maxwell and Johnson 2000). Leaf stomatal conductance was measured from the same leaves using the LCi portable photosynthesis system (ADC BioScientific Ltd., Hoddesdon, UK). Green and healthy leaves were harvested from each plant at 6 weeks after transplanting and immediately frozen in liquid nitrogen. The frozen leaves were ground into fine powder and stored in a −80 °C freezer until further analysis, as described below.

##### Chlorophyll a, Chlorophyll b, and Carotenoid Assays

Leaf tissue chlorophylls a and b and carotenoids were determined as described by Lichtenthaler [20]. In brief, 0.2 g of ground leaf tissue was homogenized in 2 mL of 80% acetone. The mixture was centrifuged at 15,000× *g* for 15 min, and the supernatant was diluted in a ratio of 1:1 with 80% acetone before measuring the absorbance (A) at 646.8, 663.2, and 470 nm using an Evolution Pro UV-vis spectrophotometer (Thermo Fisher Scientific, CA, USA) against 80% acetone as a blank. Chlorophylls (Chl) and carotenoid contents were expressed as µg g^−1^ fresh weight as follows:

(1) Chl a=12.25×A663.2−2.79×A646.8

(2) Chl b=21.50∗A646.8−5.1∗A663.2

(3) $$Carotenoid=\frac{\left[\left(1000*A470)-(1.8*Chl\;a)-(85.02*Chl\;b\right)\right]}{198}$$

##### Total Soluble Sugar and Protein Assays

The total soluble sugar was determined using the phenol-sulphuric acid procedure described by Dubois, et al. [21]. In brief, 0.2 g of ground leaf tissue was mixed with 10 mL of 90% ethanol and incubated in a water bath at 60 °C for 60 min. The volume of the mixture was re-adjusted to 10 mL with 90% ethanol and centrifuged at 12,000× *g* for 3 min. A total of 1 mL of the supernatant was thoroughly mixed with 1 mL of 5% phenol in a thick-walled glass test tube, and 5 mL of concentrated sulphuric acid was added. The reaction mixture was vortexed for 20 s and incubated in the dark for 15 min before cooling to room temperature of about 22 °C. The absorbance was read at 490 nm against a blank using a UV-vis spectrophotometer. Total sugar content was estimated using a sugar standard curve and expressed as µg of glucose g^−1^ fresh weight. Leaf tissue total protein content was determined by mixing 0.2 g of sample with 1.8 mL ice-cold extraction buffer (i.e., 50 mM potassium phosphate buffer (pH 7.0), 1% polyvinylpyrrolidone, and 0.1 mM ethylenediaminetetraacetic acid (EDTA), and centrifuged at 12,000× *g* for 20 min at 4 °C. The supernatant, a crude enzyme extract, was transferred into a new vial, and 1 mL of Bradford’s reagent was added. The absorbance of the mixture was read at 595 nm after 5 min using a UV-vis spectrophotometer, and the protein content was calculated from a bovine serum albumin standard curve ranging from 200 to 900 µg mL^−1^ [22].

##### Total Phenolics and Flavonoids Assays

The total phenolics were determined using the Folin-Ciocalteu method as described by Ainsworth and Gillespie [23], with a slight modification. In brief, 0.2 g of ground leaf tissue was homogenized in 2 mL of ice-cold 95% methanol and incubated in the dark at room temperature for 48 h. The mixture was centrifuged at 12,000× *g* for 5 min, and 100 µL of supernatant was added to 200 µL of 10% (*v*/*v*) Folin-Ciocalteau reagent and vortexed for 5 min. A total of 800 µL of 700 mM sodium carbonate was added to the mixture and incubated in the dark at room temperature for 2 h. The absorbance was read at 765 nm against a blank using a UV-vis spectrophotometer. The total phenolics were estimated using a gallic acid standard curve and expressed as mg gallic acid equivalents per g fresh weight. The total flavonoid was determined by the colorimetric method [24]. In brief, 0.2 g of ground leaf tissue was mixed with 2 mL of ice-cold 95% methanol and centrifuged at 15,000× *g* for 10 min. A total of 500 µL of supernatant was added to a reaction mixture containing 1.5 mL of 95% methanol, 0.1 mL of 10% aluminum chloride, 0.1 mL of 1 M potassium acetate, and 2.8 mL of distilled water. The mixture was incubated at room temperature for 30 min, and the absorbance was measured at 415 nm against a blank without aluminum chloride using a UV-vis spectrophotometer. Total flavonoid was estimated using a quercetin standard curve and expressed as µg quercetin g^−1^ fresh weight.

##### Hydrogen Peroxide and Lipid Peroxidation Assays

The hydrogen peroxide (H_2_O_2_) content of the leaf tissues was determined using the method of Alexieva, et al. [25]. In brief, 0.2 g of ground leaf tissue was homogenized in 1.8 mL of 0.1% (*w*/*v*) trichloroacetic acid (TCA) and centrifuged at 16,000× *g* at 4 °C for 10 min. A total of 200 μL of supernatant was added to 200 μL of 100 mM potassium phosphate buffer (pH 7) and 800 μL of 1 M potassium iodide. The reaction was incubated in the dark for 60 min before measuring the absorbance at 390 nm using a H_2_O_2_ standard curve. Lipid peroxidation was determined by leaf tissue malondialdehyde (MDA) content using the method described by Hodges, et al. [26], with slight modifications. In brief, 0.2 g of ground leaf tissue was mixed with 1.8 mL of 0.1% (*w*/*v*) TCA and centrifuged at 16,000× *g* for 10 min at 4 °C. A total of 500 µL of the supernatant was added to an equal volume of 0.5% thiobarbituric acid in 20% TCA. The reaction mixture was then incubated at 95 °C for 30 min, cooled on ice, and centrifuged at 12,000× *g* for 5 min. The absorbance of the resultant mixture was measured using a UV-vis spectrophotometer at 532 nm and non-specific absorption at 600 nm. The MDA content was estimated from the extinction coefficient of 155 m/M cm and expressed as nmol MDA g^−1^ fresh weight.

##### DPPH Free Radical Scavenging Capacity Assay

The 2,2-diphenyl-1-picrylhydrazyl (DPPH) radical scavenging capacity was determined using the method described by Dudonné, et al. [27] with a slight modification. In brief, 0.2 g of ground leaf tissue was homogenized with 1.5 mL of pure methanol and centrifuged at 12,000× *g* for 10 min. A 100 µL of the supernatant was mixed with 2.9 mL of 60 µM fresh DPPH methanolic solution, and the resultant mixture was incubated in the dark at room temperature. After 30 min, the absorbance was measured at 515 nm against a methanol blank using a UV-vis spectrophotometer. Radical scavenging activity was determined as follows: Inhibition (%)=[Ab−As)/Ab]×100%, where Ab is the blank absorbance and As is the sample absorbance.

#### 2.3.2. Phytohormone Analysis

A number of compounds namely dihydrophaseic acid (DPA), abscisic acid glucose ester (ABAGE), phaseic acid (PA), 7′ hydroxy-abscisic acid (7′OH-ABA), neo-PA, trans-ABA, and N-(Indole-3-yl-acetyl)-glutamic acid (IAA-Glu) were synthesized and prepared at the National Research Council of Canada (NRCC), Saskatoon, SK, Canada; ABA, N-(Indole-3-yl-acetyl (IAA), IAA-leucine (IAA-Leu), IAA-alanine (-Ala), IAA-aspartic acid (-Asp), zeatin (Z), zeatin riboside (ZR), isopentenyladenine riboside (iPR), isopentenyladenine (iP), indole-3-butyric acid (IBA), kinetin, salicylic acid (SA), jasmonic acid (JA), 1-aminocyclopropane-1-carboxylic acid (ACC), phenylisothiocyanate (PITC), trifluoroacetic acid (TFA) and triethylamine were purchased from Sigma–Aldrich; dhZ, dhZR, zeatin-O-glucoside (ZOG), JA-isoleucine (-Ile) and gibberellins (Gas) 1, 3, 4, 7, 8, 9, 19, 20, 24, 29, 44, and 53 were purchased from OlChemim Ltd. (Olomouc, Czech Republic). Deuterated forms of the hormones that were used as internal standards include: d3-DPA, d5-ABA-GE, d3-PA, d4-7′-OH-ABA, d3-neoPA, d4-ABA, d4-trans-ABA, d3-IAA-Leu, d3-IAA-Ala, d3-IAA-Asp, d3-IAA-Glu, 13C4-IBA, 2,2-d2-jasmonic acid, and 12,12,12-d3-jasmonic acid isoleucine (unpublished) were synthesized and prepared at NRCC SK according to Abrams, et al. [28], Galka, et al. [29], and Zaharia, et al. [30]. The d5-IAA was purchased from Cambridge Isotope Laboratories (Andover, MA, USA); 3,4,5,6-d4-2-hydroxybenzoic acid and 1-amino-[2,2,3,3-d4]cylopropane-1-carboxylic acid (d4-ACC) were purchased from CDN isotopes (Point-Claire, QC, Canada); d3-dhZ, d3-dhZR, d5-Z-O-Glu, d6-iPR, d6-iP, 15N4-kinetin, and d2-GAs 1, 3, 4, 7, 8, 9, 19, 20, 24, 29, 34, 44, 51, and 53 were purchased from OlChemim Ltd. (Olomouc, Czech Republic). The d4-ACC derivative, PTH-d4-ACC, was used as an internal standard. The deuterated forms of selected hormones used as recovery (external) standards were prepared and synthesized at NRCC SK. Calibration curves were created for all compounds of interest, including the ACC derivatives, phenylthiohydantoin-ACC (PTH-ACC), and PTH-d4-ACC. Quality control samples were run along with the tissue samples.

##### Hormone Quantification by HPLC-ESI-MS/MS

Analysis was performed on an ultra-performance liquid chromatography-electrospray ionisation tandem mass spectrometry (UPLC-ESI-MS/MS) utilizing a Waters ACQUITY UPLC system, equipped with a binary solvent delivery manager and a sample manager coupled to a Waters Micromass Quattro Premier XE quadrupole tandem mass spectrometer via a Z-spray interface. MassLynx™ and QuanLynx™ (Micromass, Manchester, UK) were used for data acquisition and analysis. The procedure for quantification of ABA and ABA catabolites, cytokinins, auxins, and gibberellins in the plant tissue samples was performed using a modified procedure described in Lulsdorf, et al. [31], while the quantification of ACC was performed using a modified procedure described in Chauvaux, et al. [32]. The procedure for quantification of SA and JA was performed as described in Murmu, et al. [33], with some modifications. These modifications have not been published. The quantitative analysis utilizes the Multiple Reaction Monitoring (MRM) function of the MassLynx control software (version 4.1). The resulting chromatographic traces for each analyte (phytohormone) and their respective deuterium labelled internal standard were quantified off-line using QuanLynx v4.1, where each trace was integrated, and the resulting ratio of signals (analyte/internal standard) were compared with a previously constructed calibration curve to yield the amount of analyte present (ng per sample). Calibration curves were generated from the MRM signals from standard solutions based on the ratio of the chromatographic peak area for each analyte to that of the corresponding internal standard. The quality control samples and internal standard and solvent blanks were prepared and analyzed along with each batch of tissue samples.

### 2.4. Statistical Analysis

Concentrations of the detected analytes in the above assays were calculated with IS calibration by interpolating the constructed linear regression curves of individual compounds using the analyte-to-internal standard peak area ratios measured from injections of the sample solution. All data obtained were subjected to one-way analysis of variance (ANOVA) using Minitab version 21.1 (Minitab, Inc., State College, PA, USA). Tukey’s honestly significant difference post-test was used to separate treatment means when the ANOVA indicated *p* ≤ 0.05. MS Excel and XLSTAT (Version 2022.1, Lumivero, CO, USA) were used to plot graphs and perform Pearson correlation, respectively.

## 3. Results

The elemental composition of the soils used for the present study varied depending on the level of soil MSWC residue after five years of treatment (Supplementary Table S1).

### 3.1. Plant Growth and Photosynthetic Activities

**Figure 1:**
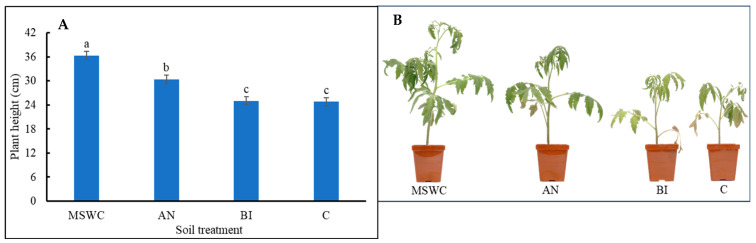
(**A**) Plant height of tomato (*Solanum lycopersicum* ‘Scotia’) plants grown in soils after 5 years of treatment with varying application frequency of municipal solid waste compost (MSWC). Soils with annual (AN), biennial (BI), and no (C) compost applications and MSWC alone. (**B**) The plants in the pots show differences in growth per treatment at 6 weeks after transplanting. Vertical lines on bars represent standard errors (N = 12), and bars with different alphabetical letters denote statistically significant differences in treatment means (N = 12) at *p* ≤ 0.05.

The trend in elemental composition was previously reported [10,17] as MSWC > AN-soil > BI-soil > C-soil. Tomato ‘Scotia’ plant growth parameters and photosynthetic indices were significantly (*p* < 0.001) influenced by differences in soil MSWC residue levels (Figure 1; Table 1). The average height of MSWC plants was ca. 16.3% higher than that of the AN-plants and ca. 31.4% higher than that of the BI-plants or C-plants (Figure 1). The plant heights for the B-Plants and C-plants were not significantly (*p* > 0.05) different. The MSWC-plants and AN plants had similarly the highest number of leaves and stem diameter at an average of ca. 63% and 3.8% more than those for the BI-plants or the C-plants, respective ely (Table 1).

All the chlorophyll fluorescence indices, i.e., minimum fluorescence yield (Fo), maximum fluorescence (Fm), variable fluorescence (Fv), and maximum quantum efficiency of PSII (Fv/Fm), except potential photosynthetic capacity (Fv/Fo), were significantly (*p* < 0.05) influenced by differences in soil MSWC residue levels (Table 1). The Fm and Fv of MSWC-plants and AN-plants were similar and significantly (*p* < 0.001) higher than those of BI-plants and C-plants. The MSWC-plants and AN-plants showed moderate but significant (*p* < 0.05) increases in Fv/Fm by ca. 4.8% and 5.4%, respectively, compared to the C-plants. Additionally, Fv/Fo of the MSWC-plants and AN-plants were significantly (*p* < 0.01) increased by ca. 16.2% and 18.8%, respectively, compared to the C-plants (Table 1). Both Fv/Fm and Fv/Fo were not significantly (*p* > 0.05) different between BI-plants and C-plants. The AN-plants recorded the highest stomatal conductance at 326.7 mmol m^−2^ s^−1^ followed by the MSWC-plants and the BI-plants at an average of 234.8 mmol m^−2^ s^−1^ (Table 1). The C-plants recorded the lowest stomatal conductance compared to the other treatments.

### 3.2. Plant Tissue Biochemical Composition

**Figure 2:**
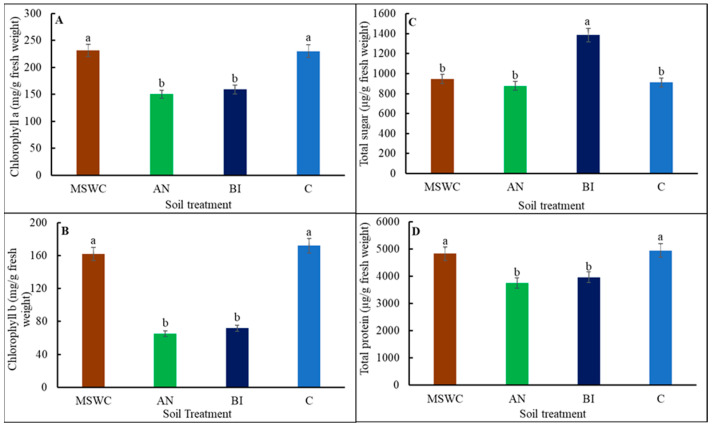
Chlorophylls a (**A**) and b (**B**), total soluble sugar (**C**), and total protein contents (**D**) of tomato (*Solanum lycopersicum* ‘Scotia’) plants grown in soils after 5 years of treatment with varying application frequency of municipal solid waste compost (MSWC). Soils with annual (AN), biennial (BI), and no (C) compost applications and MSWC alone. Vertical lines on bars represent standard errors (N = 12); bars with different alphabetical letters denote statistically significant differences in treatment means (N = 12) at *p* ≤ 0.05.

Tomato ‘Scotia’ plant leaf content of Chl a, Chl b, total protein, and total soluble sugar were significantly (*p* < 0.001) altered by the different levels of soil MSWC residue (Figure 2A–D). The trend in the variations of Chl a, Chl b and total protein contents was similar. The MSWC-plants and C-plants had higher Chl a, Chl b and total protein compared to the similar values for AN-plants and the BI-plants. Compared to the AN-plants, the BI-plants had slight but non-significant (*p* > 0.05) increases in Chl a, Chl b, and total protein contents.

**Figure 3:**
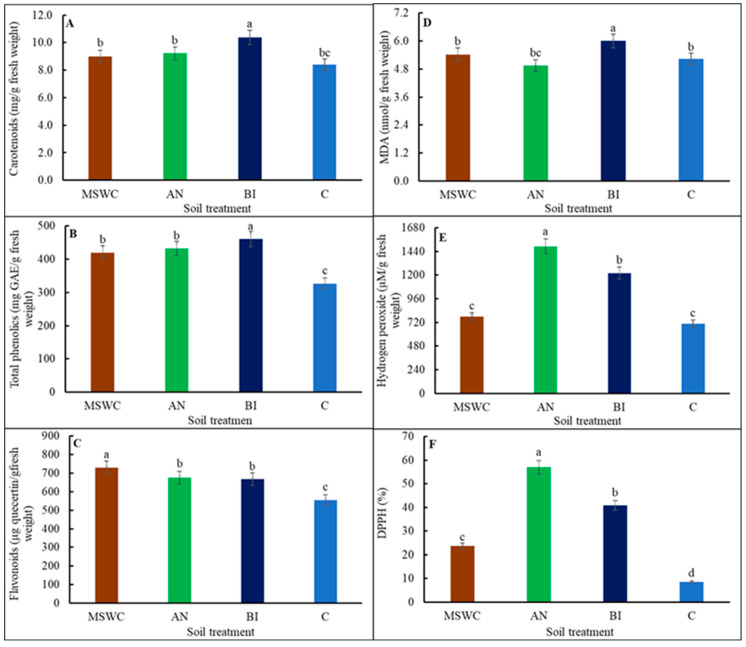
Carotenoids (**A**), total phenolics (**B**), flavonoids (**C**), malonaldehyde (MDA) (**D**), hydrogen peroxide (H_2_O_2_) (**E**) and 2,2-diphenyl-1-picrylhydrazyl (DPPH) radical scavenging capacity (**F**) of tomato (*Solanum lycopersicum* ‘Scotia’) plants grown in soils after 5 years of treatment with varying application frequency of municipal solid waste compost (MSWC). Soils with annual (AN), biennial (BI), and no (C) compost applications and MSWC alone. Vertical lines on bars represent standard errors (N = 12); bars with different alphabetical letters denote statistically significant differences in treatment means (N = 12) at *p* ≤ 0.05.

In contrast, the BI-plants had the highest total soluble sugar content, which was ca. 34.2% more than the average for MSWC-plants, AN-plants, and C-plants (Figure 2C). Furthermore, the variations in soil MSWC residue levels differentially altered the composition of tomato ‘Scotia’ plant secondary metabolites (Figure 3A–F). The trends for carotenoids and phenolics were similar (Figure 3A,B). Specifically, it was found that BI-plants had significantly (*p* < 0.05) higher carotenoids compared to MSWC-plants, AN-plants, and C-plants (Figure 3A). Similarly, total phenolics in the BI-plants were ca. 7.6% more than the average for MSWC-plants and AN-plants, or ca. 29.1% higher than that of the C-plants (Figure 3B). However, flavonoids were significantly (*p* < 0.01) increased by soils with MSWC residue, irrespective of the treatment level, with the trend being MSWC = AN = BI > C (Figure 3C). The BI-plants had ca. 11% more MDA than the average for MSWC-plants and C-plants, while the AN-plants had the lowest MDA (Figure 3D). Overall, the trends for H_2_O_2_ and DPPH were similar. For both compounds, AN-plants were superior, with a significant (*p* < 0.001) increase in H_2_O_2_ by ca. 18%, 47.8%, and 52.5% compared to BI-plants, MSWC-plants, and C-plants, respectively (Figure 3E), and an increase in DPPH by ca. 28.5%, 58.6%, and 85% compared to BI-plants, MSWC-plants, and C-plants, respectively (Figure 3F).

### 3.3. Phytohormone Composition

**Figure 4:**
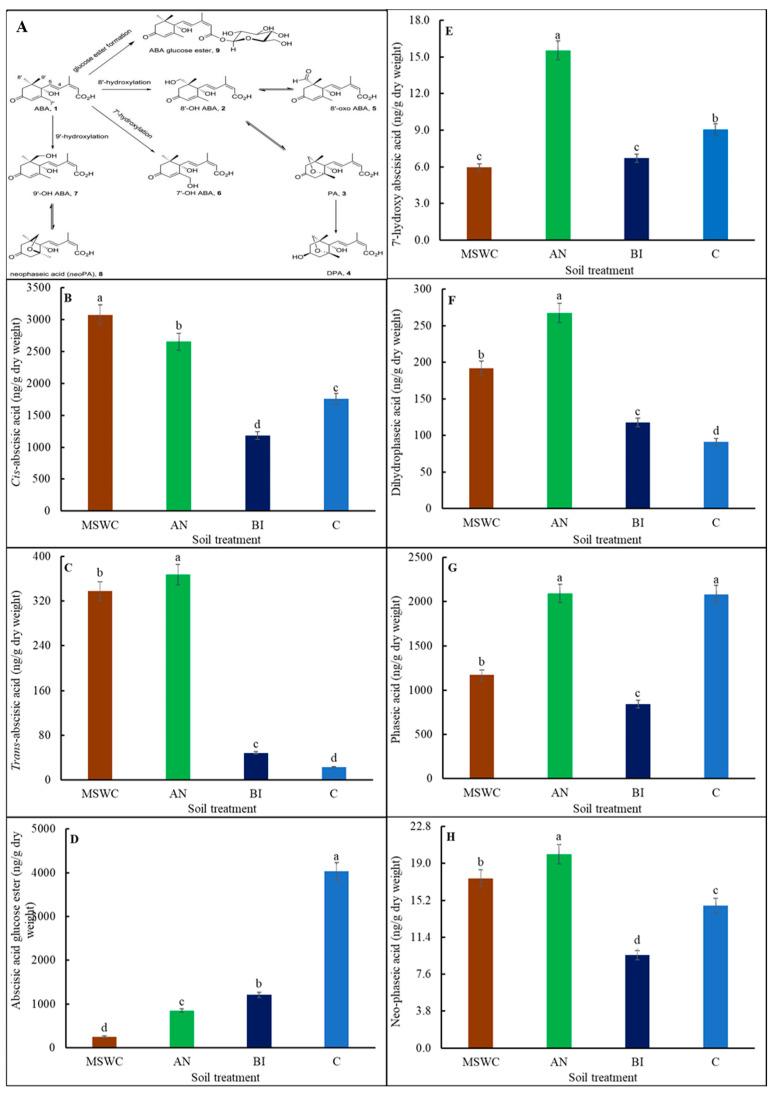
Abscisic acid (ABA) pathway (**A**), cis-ABA (**B**), trans-ABA (**C**), abscisic acid glucose ester (ABAGE) (**D**), 7′hydroxy-abscisic acid (7′OH-ABA) (**E**), dihydrophaseic acid (DPA) (**F**), phaseic acid (PA) (**G**), and neo-PA (**H**) contents of tomato (*Solanum lycopersicum* ‘Scotia’) plants grown in soils after 5 years of treatment with varying application frequency of municipal solid waste compost (MSWC). A Soils with annual (AN), biennial (BI), and no (C) compost applications and MSWC alone. Vertical lines on bars represent standard errors (N = 12); bars with different alphabetical letters denote statistically significant differences in treatment means (N = 12) at *p* ≤ 0.05.

The hormones ABA and its catabolites, cytokinins, auxins, gibberellins, ACC, and free and conjugated JA and SA were not detected in soils with or without MSWC residue (Supplementary Table S2). However, the compositions of detected endogenous plant hormones were markedly altered by soil MSWC residue levels (Supplementary Table S3). The ABA pathways in Figure 4A indicate that the conjugation of ABAGE and 8′-hydroxylation and the formation of PA with subsequent reduction to DPA were the predominant catabolic pathways. The results showed that the accumulation of cis-ABA was significantly (*p* < 0.01) increased in MSWC-plants by ca. 13.8%, 61.5%, and 42.8% compared to AN-plants, BI-plants, and C-plants, respectively (Figure 4B). The trend for trans-ABA was AN-plants > MSWC-plants >> BI-plants > C-plants (Figure 4C).

Comparatively, the accumulation of cis-ABA in the plants was much higher than trans-ABA. Conversely, the higher the soil MSWC residue level, the less the ABAGE accumulation in the plants, i.e., (Figure 4D). Compared to C-plants, ABAGE was reduced by ca. 93.7%, 78.9%, and 70% in MSWC-plants, AN-plants, and BI-plants, respectively. On the other hand, AN-plants had significantly (*p* < 0.001) the highest 7′OH-ABA, which was ca. 62.5% compared to C-plants and ca. 59.4% compared to MSWC-plants or BI-plants (Figure 4E). The difference in 7′OH-ABA between MSWC-plants and AN-plants was not significant (*p* > 0.05). Likewise, the level of soil MSWC residue significantly (*p* < 0.01) influenced DPA, PA, and neo-PA contents, which were consistently higher in the AN-plants compared to the other treatments (Figure 4F–H).

The AN-plants had ca. 28.1%, 55.8%, and 65.9% more DPA (Figure 4F); ca. 44%, 59.7%, and 0.05% more PA (Figure 4G); and ca. 15%, 50%, and 25% more neo-PA (Figure 4H) compared to MSWC-plants, BI-plants, and C-plants. There was no significant (*p* > 0.05) difference in PA between the AN-plants and the C-plants. The BI-plants had the lowest PA and neo-PA, while the C-plants had the lowest DPA.

**Figure 5:**
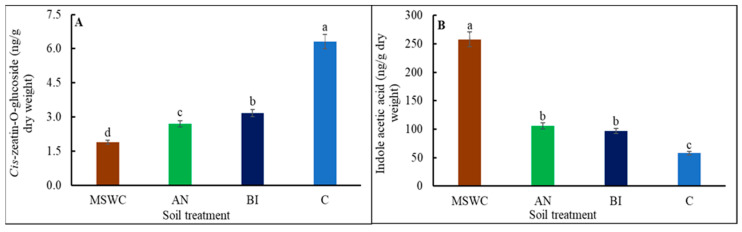
Cis-zeatin-O-glucoside (**A**) and indole acetic acid (**B**) contents of tomato (*Solanum lycopersicum* ‘Scotia’) plants grown in soils after 5 years of treatment with varying application frequency of municipal solid waste compost (MSWC). Soils with annual (AN), biennial (BI), and no (C) compost applications and MSWC alone. Vertical lines on bars represent standard errors (N = 12), bars with different alphabetical letters denote statistically significant differences in treatment means (N = 12) at *p* ≤ 0.05.

Bioactive free base cytokinins, i.e., cis- and trans-zeatin, dihydrozeatin, and isopentenyladenine, were not detected in the tomato ‘Scotia’ plants despite differences in soil MSWC residual levels (data not presented). However, the biosynthetic precursors of zeatin ribosides, i.e., both cis- and trans-ZR and iPR, were only found in the MSWC plants and the AN-plants (Supplementary Table S3). KIN was also detected, but only in the AN-plants. Quantitatively, cis-ZR, trans-ZR, and iPR were, respectively, ca. 97.8%, 94.8%, and 66.7% higher in the MSWC-plants compared to the AN-plants. Also, Cis-ZOG was the only cytokinin that was detected in all the plants with the trend, MSWC-plants < AN-plants < BI-plants < C-plants (Figure 5A). Thus, high levels of soil MSWC residue reduced cis-ZOG accumulation in MSWC-plants, AN-plants, and BI-plants by more than ca. 50% to 66.7% compared to C-plants.

**Figure 6:**
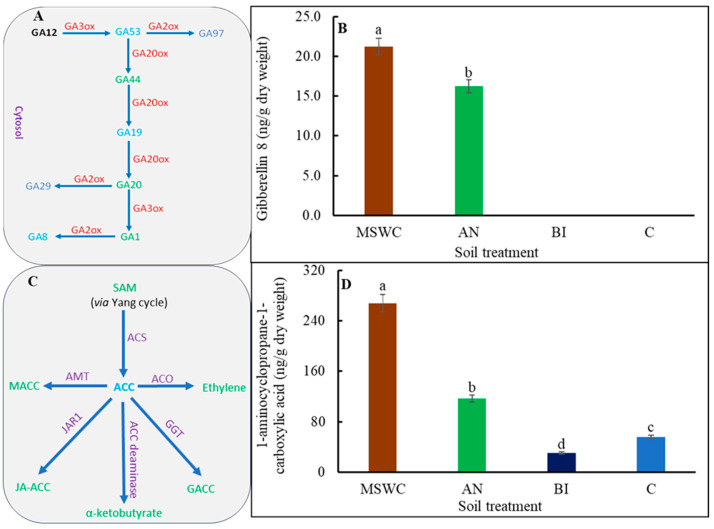
Gibberellic acid (**A**) and ethylene (**C**) pathways and gibberellins 8 (**B**) and 1-aminocyclopropane-1-carboxylic acid (**D**) contents of tomato (*Solanum lycopersicum* ‘Scotia’) plants grown in soils after 5 years of treatment with varying application frequency of municipal solid waste compost (MSWC). Soils with annual (AN), biennial (BI), and no (C) compost applications and MSWC alone. Vertical lines on bars represent standard errors (N = 12); bars with different alphabetical letters denote statistically significant differences in treatment means (N = 12) at *p* ≤ 0.05.

A reverse trend was found in Figure 5B for IAA, which was the only auxin detected in the tomato plants under the conditions of the present study. The IAA accumulation in MSWC-plants, AN-plants, and BI-plants increased by ca. 344.8%, 101.7%, and 67.2%, respectively. Also, the only GAs detected in the plants were GA53, GA19, and GA8 (Supplementary Table S3). The three detected GAs can be found in the 13-hydroxylation pathway that starts with GA53 formation through a series of oxoglutarate-dependent enzymatic steps to form GA19 with end products of GA29 and GA8, as represented in Figure 6A. GA53, GA19, and GA8 were all reported for the MSWC-plants, while only GA8 was reported for the AN-plants, but in a relatively lower quantity (Figure 6B). No GA was detected in the BI-plants or the C-plants (Supplementary Table S3). All the plants accumulated ACC, irrespective of treatment differences (Figure 6D; Supplementary Table S3). As shown in Figure 6C, ACC can be enzymatically conjugated to form malonyl-ACC (MACC), γ-glutamyl-ACC (GACC), and jasmonoyl-ACC (JA-ACC). However, the MSWC-plants exhibited the highest ACC content, which was ca. 56.3%, 88.8%, and 79.1% higher than those of the AN-plants, BI-plants, and C-plants, respectively (Figure 6C).

**Figure 7:**
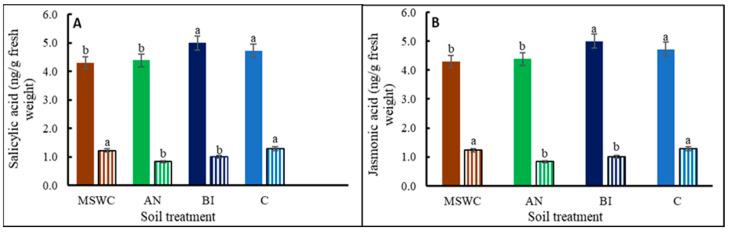
Salicylic acid (panel (**A**): solid and stripped bars are free and conjugated salicylic acids, respectively) and jasmonic acid (panel (**B**): solid and stripped bars are free and jasmonic acid-isoleucine, respectively) contents of tomato (*Solanum lycopersicum* ‘Scotia’) plants grown in soils after 5 years of treatment with varying application frequencies of municipal solid waste compost (MSWC). Soils with annual (AN), biennial (BI), and no (C) compost applications and MSWC alone. Vertical lines on bars represent standard errors (N = 12), bars with different alphabetical letters denote statistically significant differences in treatment means (N = 12) at *p* ≤ 0.05.

Free and conjugated forms of SA and JA were detected in all the tomato ‘Scotia’ plants, but their compositions significantly (*p* < 0.05) varied with the level of residual soil MSWC (Supplementary Table S3). Conjugated SA was remarkably higher than free SA, particularly in the BI-plants (Figure 7A). The conjugated SA in BI was ca. 315.7%, 124.1%, and 281.8% higher than those for MSWC-plants, AN-plants, and C-plants, respectively. The trend for free JA was BI-plants > C-plants > MSWC-plants = AN-plants, and the trend for free JA-Ile was MSWC-plants = C-plants > AN-plants > BI-plants. Conversely, free JA, which ranged from 4 to 5 ng/g, was significantly (*p* < 0.05) higher than its conjugated form, i.e., JA-Ile, which ranged between 1 and 2 ng/g (Figure 7B). Generally, the trends for the free SA and conjugated SA versus the free JA and JA-Ile were similar.

## 4. Discussion

Compost contains humic and non-humic substances, and generally, their initial release occurs at a slow rate before increasing over time. Moreover, continuous application of compost over the long term leaves residual chemical substances in the soil, which later become available to plants through various microbial and non-microbial decomposition processes. The present study demonstrated the significance of compost residue on plant growth, photosynthetic efficiency, and phytohormone and secondary metabolite accumulation in tomato ‘Scotia’ plants. The results showed that an increase in the level of soil MSWC residue increased the growth of MSWC-plants and AN-plants compared to BI-plants (moderate residue) and C-plants (no residue). This finding can be linked to increased soil residual nutrients and plant growth promoting compounds that instigated stomatal conductance and the efficiency of light absorption by chlorophyll molecules and electron transfer to increase photosynthetic efficiency and assimilate production [34,35]. Fv/Fm is a stress determinant index, and a value greater than 0.78 can be associated with healthy plants [36], as recorded for MSWC-plants and AN-plants. The health of the BI-plants was marginal, and that of the C-plants was low.

Chlorophyll is an indispensable photosynthetic pigment, but its biosynthesis and functions are affected by various climate, edaphic, and management factors [37]. The functions of Chl a and Chl b are to absorb red-orange and blue-purple lights, respectively, which were increased in both MSWC-plants and C-plants compared to the AN-plants and BI-plants. According to Wang, et al. [38], a reduction in the activity of chlorophyll-related proteins causes a concomitant reduction in Chl a and Chl b, as recorded for AN-plants and BI-plants. Furthermore, the slightly high carotenoids in AN-plants and BI-plants might have reduced light absorption capacity that led to the reductions in Chl a and Chl b as previously reported [39,40]. BI-plants also accumulated high amounts of total soluble sugar, which could be attributed to low sugar catabolism. Physiologically, it seemed the combination of low leaf chlorophylls, high total soluble sugar, and high carotenoids accumulation in BI-plants could have down-regulated photosynthesis, leading to reductions in phloem transport and plant growth [41]. The down-regulation of photosynthesis in BI-plants could also be attributed to feedback inhibition and chloroplast and nuclear coordination mechanisms [42,43], which are characterized by the maintenance of homeostasis, where high sugar levels signal the plant that there is already an ample supply of carbohydrates, thus reducing the need for further production. The C-plants were the most stunted due to many edaphic stress factors, including low soil organic matter content and poor soil fertility, as extensively captured in previous publications [10,17].

**Figure 8:**
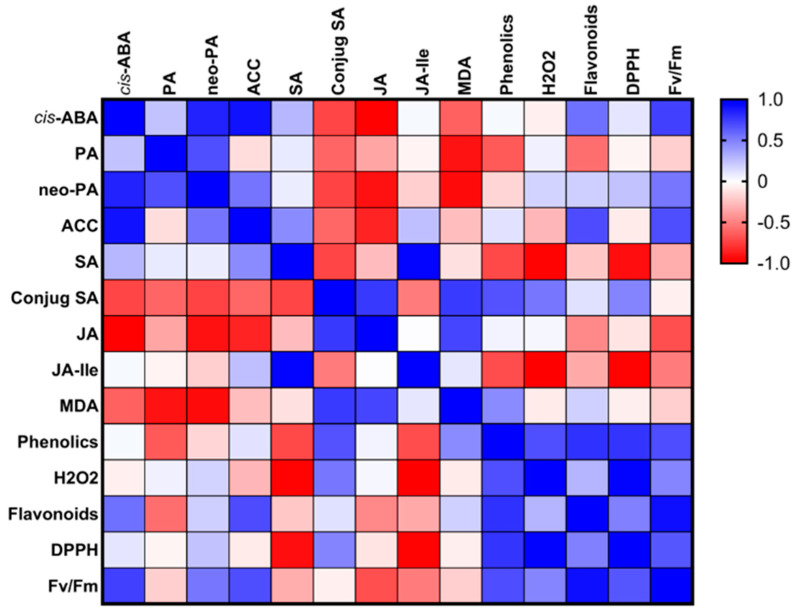
Pearson correlation matrix for maximum quantum efficiency of PSII, secondary metabolites, and phytohormones as affected by residual municipal solid waste compost (MSWC) after 5 years of application. The blue sections indicate positive correlation, and the red sections indicate negative correlation. The shade of white indicates no correlation.

Phenolics and flavonoids play key plant protective roles by mitigating photo-oxidative damage and providing defense against abiotic and biotic stresses [8]. These compounds were similarly increased in all the plants that were grown in soils with MSWC residue compared to C-plants. MDA is an indication of lipid peroxidation of PUFAs in plant membranes in response to oxidative stress, and as such, a continuous increase in MDA can potentially alter and deactivate PSII core proteins and Rubisco [44]. Therefore, the high level of MDA in BI-plants suggests a high loss of membrane integrity and modification of PSII core proteins and RUBISCO [45]. BI compost application could have resulted in rapid nutrient depletion, leading to plant starvation like the control. Under such nutrient stress, the plant undergoes numerous physiological and biochemical processes for survival and adaptation. Again, this imbalance, in addition to an increase in MDA, can reduce stomatal conductance and potential photosynthetic capacity, as observed in BI-plants and C-plants (Figure 8; [46]). Another stress response compound is H_2_O_2_, which serves two functions. H_2_O_2_ is a reactive oxygen species (ROS) that can damage many cellular structures as well as function as a signaling molecule for the regulation of various physiological and biochemical processes [7]. It can be surmised that an increase in H_2_O_2_ may have increased photosynthetic activities, total phenolics, and flavonoids in AN-plants but not in BI-plants. The high H_2_O_2_ in AN-plants seconded by BI-plants can be associated with high DPPH radical scavenging activities (Figure 8), which suggests increased cellular activities by antioxidant enzymes to protect the plant from ROS [9].

Although plant growth hormones were previously reported in compost [16], there were no detectable hormones in the 5-year-piled MSWC or the treated and control soils used in the present study. This proves the inducement of plant endogenous hormone production by soil residual compost, especially in plants grown in MSWC alone or AN-soil. We did not detect residual hormones in the soil; the complex interactions between compost-derived compounds, soil microorganisms, and plants likely contributed to the observed changes in plant hormone levels. The compost-treated soil could also influence hormone levels through nutrient availability, which is likely to influence plant hormone synthesis and signaling [47]. MSWC application can contribute to improved soil structure, which can enhance water retention, potentially reducing plant stress [48,49]. This improved growing environment could indirectly affect hormone levels, particularly stress-related hormones like ABA [6,50,51]. Also, the addition of compost can significantly alter the soil microbiome [10]. These microorganisms can produce plant growth-promoting substances, including hormone-like compounds, or influence endogenous hormone production through various signaling pathways [52,53]. Further research approaches, including metabolomics and transcriptomics, could ultimately provide a clear understanding of these intricate relationships in the plant-soil system.

Usually, hormones crosstalk with each other to increase the survival of plants under stress conditions [7,54]. Specifically, cis- and trans-ABA are formed as products of the isomerization of endogenous ABA, and while cis-ABA is biologically active, trans-ABA is biologically inert [55]. Cis-ABA stimulates anthocyanin biosynthesis, which has been shown to play a vital antioxidant role in protecting plants against ROS damage [7,55]. As such, the high cis-ABA in MSWC-plants and AN-plants suggest enhancement of cellular adaptive immunity that culminated in increased photosynthetic capacity and plant growth as shown in the Pearson correlation chart in Figure 8. The protective mechanisms include delay of leaf senescence, provision of photoprotection, reduction in damage to photosystem II, and suppression of photorespiration [56,57]. ABAGE, the stored form of ABA, was high in C-plants but low in MSWC-plants, followed by AN-plants, and the lowest in BI-plants. Thus, a large proportion of ABA was metabolized into ABAGE in plants that were relatively stressed and stunted due to lower levels of soil MSWC residue, as reported by Vernieri, et al. [58]. DPA is an inactive precursor of PA, which is a biologically active form of ABA through the action of the 9-cis-epoxycarotenoid dioxygenase enzyme [59]. Neo-PA is a metabolite of PA and may have a role in the regulation of ABA levels in plants. It is, therefore, obvious that soil MSWC residue moderated ABA homeostasis and signal transduction for efficient physiological processes as observed in the AN-plants compared to the others.

IAA is an auxin known to promote cell division and cell elongation [60]. Therefore, the trend in tomato ‘Scotia’ plant growth parameters can be associated with the observed trend in plant endogenous IAA contents i.e., MSWC-plants > AN-plants > BI-plants > C-plants. IAA and cZOG (cytokinin) are two major phytohormones with opposing effects on plant growth and development [2,7]. In the present study, the increase in IAA was accompanied by a reduction in cZOG and vice versa. Additionally, GA8 promotes cell elongation [61], and the higher the soil level of residual MSWC, the higher the GA8 content in the plants. ACC is a non-protein amino acid and a direct precursor of ethylene, and both compounds actively participate in a wide range of plant growth, developmental, and defense programs [62,63]. The relatively higher ACC content of the MSWC-plants followed by the AN-plants can explain their higher growth components, which is consistent with the role of ethylene in plant growth [64]. SA is a phenolic compound and a signaling molecule that plays a critical role in plant growth, development, and abiotic stress defense [7]. It was evident that free SA underwent various biologically relevant chemical alterations to render it inactive in its conjugated form in the plants [65]. JA, produced from α-linolenic acid, plays a significant role in regulating biotic and abiotic stress in plants [7]. JA can conjugate with isoleucine to form JA-Ile [66], which is the active form of JA in plants. Therefore, the significant accumulation of JA-Ile in both MSWC-plants and C-plants can be linked to the observed increase in their total protein contents compared to the AN-plants and the BI-plants. Several studies have revealed that total protein content can modulate JA-Ile levels through its influence on the synthesis, degradation, and signaling pathways of JA, creating a dynamic relationship between protein levels and JA-Ile activity [67,68,69]. While some studies demonstrated an antagonistic relationship between JA and SA [70], many others have shown a synergistic relationship with SA involved in the early stress response while the JA response was at the later stage of stress [54,71].

## 5. Conclusions

**Figure 9:**
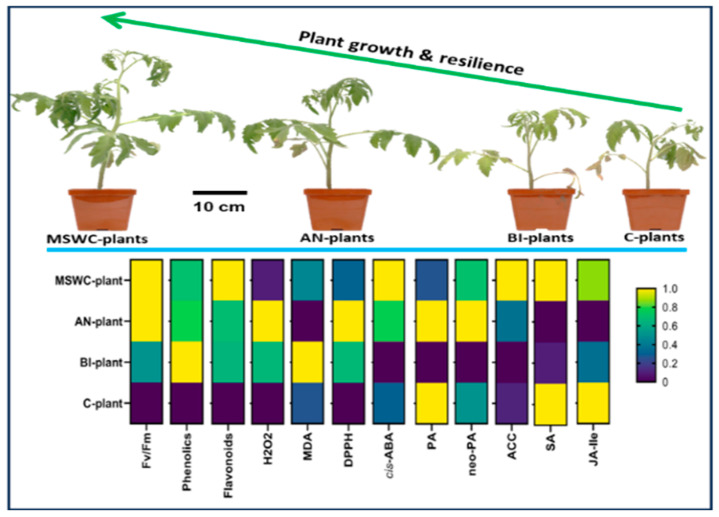
Heatmap summary of tomato (*Solanum lycopersicum* ‘Scotia’) plant growth, photosynthetic activity, and abiotic stress-response secondary metabolites and phytohormones as affected by residual municipal solid waste compost (MSWC) after 5 years of application. Annual (AN), biennial (BI), and no (C-soil) compost applications and MSWC alone.

The present study shows increases in plant growth and photosynthetic activity under high levels of soil compost residue, which is consistent with our previous observations using the same soil [10,17]. We determined that compost residue stimulates plants to increase photosynthetic activity and to accumulate significant amounts of known abiotic stress indicators MDA and H_2_O_2_ and abiotic stress mitigating compounds SA, ACC, and ABA catabolites—cis-ABA, PA, and neo-PA coupled with enhanced DPPH radical scavenging activities as shown in Figure 9. This supports our hypothesis that residual compost has biostimulatory effects that can promote normal cellular balance through homeostasis and the alteration of specific metabolic pathways and hormonal activities under abiotic stress conditions. Phytohormones have distinct functions, and their interactions involve complex crosstalk where they either work synergistically or antagonistically to accumulate significant amounts of abiotic stress-mitigating compounds that increase plant resilience and adaptation programs, which was apparent in the present study. These results can be adopted on-farm to boost plant tolerance to abiotic stress on both organic and conventional farms. Future work should investigate the flow of these stress-response compounds in plants under abiotic stress conditions.

## Figures and Tables

**Table 1:** Plant growth components, chlorophyll fluorescence indices, and stomatal conductance of tomato (*Solanum lycopersicum* ‘Scotia’) grown in soils after 5 years of treatment with varying application frequency of municipal solid waste compost (MSWC). Annual (AN-soil), biennial (BI-soil), and no (C-soil) compost applications and MSWC alone. Values with different alphabetical letters denote statistically significant differences in treatment means (N = 12) at *p* ≤ 0.05.

Treatments	Number of Leaves	Stem Diameter (mm)	Fo	Fm
**MSWC-soil**	8.5a	7.1a	213.5a	1112.9a
**AN-soil**	7.8a	6.6a	204.3a	1092.9a
**BI-soil**	5.0b	5.5b	203.8a	986.3b
**C-soil**	5.0b	4.8b	224.6a	984.9b
***p*-value**	0.00	0.001	0.09	0.00
**Treatments**	**Fv**	**Fv/Fm**	**Fv/Fo**	**Stomatal Conductance (mmol m^−2^ s^−1^)**
**MSWC-soil**	899.4a	0.808a	4.215ab	229.6b
**AN-soil**	888.6a	0.813a	4.353a	326.7a
**BI-soil**	777.4b	0.793b	3.837bc	240.0b
**C-soil**	761.6b	0.769b	3.534c	212.9c
***p*-value**	0.00	0.04	0.004	0.05

## Data Availability

The data presented in this study are available on request from the corresponding author due to privacy.

## Supplementary Materials

The following supporting information can be downloaded at: https://www.mdpi.com/article/10.3390/metabo14080400/s1, Table S1: Residual chemical element concentration of municipal solid waste compost and soils applied with the compost at different frequencies for 5 years; Table S2: Plant hormones in soils treated with municipal solid waste compost at different application frequencies.; Table S3: Plant hormones in tomato (*Solanum lycopersicum* L. ‘Scotia’) as influenced by soils treated with municipal solid waste compost at different application frequencies.
